# Sensitivity of non-conditional climatic variables to climate-change deep uncertainty using Markov Chain Monte Carlo simulation

**DOI:** 10.1038/s41598-022-05643-8

**Published:** 2022-02-02

**Authors:** Babak Zolghadr-Asli, Omid Bozorg-Haddad, Maedeh Enayati, Hugo A. Loáiciga

**Affiliations:** 1 Department of Irrigation and Reclamation Engineering, Faculty of Agricultural Engineering and Technology, College of Agriculture and Natural Resources, University of Tehran, Karaj, 3158777871 Iran; 2 Department of Geography, University of California, Santa Barbara, CA 93106 USA

**Keywords:** Climate sciences, Ecology, Environmental sciences, Hydrology, Natural hazards, Solid Earth sciences, Engineering, Mathematics and computing

## Abstract

There is substantial evidence suggesting climate change is having an adverse impact on the world’s water resources. One must remember, however, that climate change is beset by uncertainty. It is therefore meaningful for climate change impact assessments to be conducted with stochastic-based frameworks. The degree of uncertainty about the nature of a stochastic phenomenon may differ from one another. Deep uncertainty refers to a situation in which the parameters governing intervening probability distributions of the stochastic phenomenon are themselves subjected to some degree of uncertainty. In most climatic studies, however, the assessment of the role of deep-uncertain nature of climate change has been limited. This work contributes to fill this knowledge gap by developing a Markov Chain Monte Carlo (MCMC) analysis involving Bayes’ theorem that merges the stochastic patterns of historical data (i.e., the prior distribution) and the regional climate models’ (RCMs’) generated climate scenarios (i.e., the likelihood function) to redefine the stochastic behavior of a non-conditional climatic variable under climate change conditions (i.e., the posterior distribution). This study accounts for the deep-uncertainty effect by evaluating the stochastic pattern of the central tendency measure of the posterior distributions through regenerating the MCMCs. The Karkheh River Basin, Iran, is chosen to evaluate the proposed method. The reason for selecting this case study was twofold. First, this basin has a central role in ensuring the region’s water, food, and energy security. The other reason is the diverse topographic profile of the basin, which imposes predictive challenges for most RCMs. Our results indicate that, while in most seasons, with the notable exception of summer, one can expect a slight drop in the temperature in the near future, the average temperature would continue to rise until eventually surpassing the historically recorded values. The results also revealed that the 95% confidence interval of the central tendency measure of computed posterior probability distributions varies between 0.1 and 0.3 °C. The results suggest exercising caution when employing the RCMs’ raw projections, especially in topographically diverse terrain.

## Introduction

Uncertainty refers to a situation in which the knowledge about the past, present, or more commonly, the future is not absolute. However, the degree of uncertainty is, also, not an absolute term. The amount of uncertainty may differ from one phenomenon to another to the point that it is often described as a *spectrum of uncertainty*^1^. In some cases these uncertainties might be mild and may only concerned with minor details; in others, they may be severe uncertainties that underlie all aspects of the phenomena of interest^2^. For instance, in a given case, we may know that a given variable would always be within a specified range. More importantly, the stochastic nature of this phenomenon can be described mathematically through probability distributions. In other cases, however, we might not have a grasp on the plausible range of the variable, nor is there any explicit mathematical formulation about the stochastic nature of the said phenomenon. Evidently, we are technically dealing with uncertainty in both cases, but the level of uncertainty in the latter case is clearly more pronounced than in the former case. In the spirit of acknowledging the multistage nature of uncertainty, the void between two absolute conditions of knowing and not knowing can be divided into five different levels of uncertainty.

Level one uncertainty describes a clear future; however, in these cases one must also acknowledge the presence of a mild, yet, implicitly expressed source of uncertainty. Conducting a conventional sensitive analysis would suffice to address this degree of uncertainty. Level two uncertainty can be handled by representing the stochastic nature of the phenomenon with a single probability distribution function (*pdf*). One cannot be sure about a specific outcome but there is a certain degree of confidence about the nature of the outcomes’ probabilistic behavior. In level three uncertainty, we face a set of discrete alternative outcomes, each of which is associated with a specific probability. There is no information available regarding the likelihood of observing the feasible stochastic continuous alternatives in level four uncertainty. Finally, level five uncertainty means the only certain thing is that nothing is for certain, and while no further information can be provided about the uncertain nature in this instance, the very act of acknowledging this uncertainty has its own merits. Some of the ironies that arise with the occurrence of uncertain events are explained by Taleb’s^3^ *black swan theory*. In this case the decision-makers are mainly concerned with those highly improbable, yet entirely plausible, events that are out of the realm of conveniently expected outcomes. This notion may be considered as a reversion of the hot-hand fallacy, whereby observing a strike of successful outcomes may deceive the decision-maker into underestimating the probability of facing a failure event^4^.

The fourth and fifth levels of the uncertainty spectrum constitute *deep uncertainty*^1^. As one explores further into the higher stages of uncertainty, the conventional statistical viewpoints require specific revisions to capture the nature of uncertainty. In the context of deep uncertainty, for instance, one cannot merely represent the stochastic nature of the problem using a singular *pdf*, for in such cases the parameters used to define these *pdf*s are themselves uncertain. In lower stages of uncertainty it is possible to treat these parameters as deterministic values even though they behave in a stochastic manner in the context of deep uncertainty.

As far as addressing, and, in turn, understanding the uncertain nature of stochastic phenomena is concerned, Bayes’ theorem has, repeatedly, proven to be an effective tool for the job. According to Bayesian theorem, statistical information gathered from a sample is merged and, in turn, used to modify the prior knowledge about a stochastic phenomenon. The byproduct of such a process is a more informed description of the stochastic nature of the process (i.e., the posterior distribution).

The Bayesian theorem has been used on many occasions to address the uncertainties of climatic phenomena (e.g.,^5–7^). Yet, applying this method in practice is not without its challenges, chief among which is the computational difficulty of this method^8^. In the late 1950s, innovative and pragmatic viewpoints of Ulam and von Neumann, who theorized the early concept of the Monte Carlo method, paved the way for incorporating Bayesian theorem into complex real-world problems^9^. From that point onward, Monte Carlo simulation and its hybrid advanced variations, such as Markov Chain Monte Carlo (MCMC) have been instrumental in addressing the uncertainty in various fields of study, including but not limited to climatic studies (e.g.,^10–13^).

In the context of climatic science, however, the most notable source of uncertainty is perhaps the climatic change phenomenon^14^. Accordingly, the United Nations Framework Convention on Climate Change (UNFCCC) defines *climate change* as follows^14^: “*a change of climate which is attributed directly or indirectly to human activity that alters the composition of the global atmosphere and which is in addition to natural climate variability observed over comparable time periods*.” The primary outcome of plausible changes in climate is a rise in global average air temperature (i.e., global warming); yet, the impacts of this phenomenon on a regional scale and various time scales are indeed uncertain.

MCMC simulation has been used on many studies to address the uncertainties that are associated with climate change. Nawaz and Adeloye^15^, for instance, carried out a Monte Carlo based simulation to investigate the impact of several global circulation models (GCMs) on water resources yield in northeastern England. As a result, they were able to determine a range of possible streamflow values for different models and time-periods. Kwon et al.^16^ utilized a weather state-based, stochastic multivariate model as a conditional probability model for simulating precipitation. The latter authors coupled a Bayesian MCMC scheme with their precipitation-runoff model. Wang and Wang^17^ coupled a convection-permitting climate model with MCMC simulation to generate high-resolution climate projections over Texas, USA. These authors were able to provide estimates with a 95% confidence interval of projected future of precipitation, potential evaporation, and streamflow.

The uncertainty is of utmost importance but it has not been considered in many recent environmental studies (e.g.,^18–23^). The cited studies were limited to addressing the third level of uncertainty. Such studies tapped the concept of uncertainty by presenting the stochastic pattern of climatic variables; nevertheless, there is still a gap in addressing the deep uncertainty that is associated with the climate change phenomenon. This study, thus, proposes an MCMC based framework to extract the posterior distribution of the non-conditional climatic variables, which is the average surface air temperature, under climate change conditions and to shed light on the stochastic nature of the posterior *pdf*s’ computed parameters by providing a separate *pdf* for their central measure tendency. The Karkheh River Basin, Iran, is selected to demonstrate the proposed framework due to its role in ensuring the water, food, and energy security of many regions with diverse topographic profiles, which impose a challenge for most regional climate models (RCMs).

## Materials and methods

There may be evidence supporting the probabilistic nature of stochastic phenomena,, which is commonly represented as a probability distribution function (i.e., the *prior distribution*). However, additional information can help decision-makers redefine and alter their previous viewpoints toward the stochastic nature of the given phenomenon (i.e., the *likelihood function*). Naturally, if the previous perception about the event matches with the additional gathered information, it would be safe to assume that the pre-exciting opinion agrees with the actual stochastic nature of the phenomenon. If the likelihood function, however, does not closely resemble the prior distribution, the Bayesian theorem enables the decision-makers to merge these pieces of information into a more realistic description of the phenomenon (i.e., the *posterior distribution*). In general, the above-stated theorem can be mathematically expressed as follows (see, e.g.,^24^):

1 $$ p(\theta |\xi ) = \frac{p(\theta ) \times p(\xi |\theta )}{{p(\xi )}} $$

in which, *θ*  is the  set of unknown variables (say, parameters); *ξ*  is the set of additional information; $p(\theta |\xi )$ is the posterior distribution; $p(\theta )$ is the prior distribution; $p(\xi |\theta )$ is the likelihood function; and $p(\xi )$ is the evidence function that acts as a normalization constant. From a practical point of view the evidence function may be recast as a proportionality constant, and, thus, the Bayesian theorem is rewritten as follows^17^:

2 $$ p(\theta |\xi ) \propto p(\theta ) \times p(\xi |\theta ) $$

The Bayesian theorem seems a promising concept in theory, yet, it may require a large computational effort in some applications. Markov Chain Monte Carlo (MCMC) sampling, however, provides practical solution to circumvent the computational burden associated with the implementation of the Bayesian theorem. The core idea of MCMC is to generate a large enough sample of the posterior distribution. There are various MCMC sampling algorithms, the Metropolis–Hastings algorithm^25,26^ being a well-established and efficient alternative. Although the Metropolis–Hastings algorithm was theorized a while back it remains one of the most popular and accurate ways to implement the Bayesian theorem (e.g.,^27–31^).

The Metropolis–Hastings algorithm employs an acceptance/rejection principle to conduct a random walk through the parameter space and generate a chain of values that constitute a large sample from the posterior distribution. The pseudo code for the Metropolis–Hastings algorithm is as follows^25,26^:

Step I: Initiate the iterative algorithm (*i* = 0) by randomly selecting a start point in the parameter space denoted by $\theta_{i}$.
Step II: Randomly chose an alternative point ($\theta^{*}$) in the parameter space within the vicinity of $\theta_{i}$. Evaluate the acceptance probability (*α*) to control whether to accept or reject the alternative point ($\theta^{*}$), using the following equation:
  3 $$ \alpha_{i} = \min \left\{ {1,\frac{{p(\theta^{*} ) \times p(\xi |\theta^{*} )}}{{p(\theta_{i} ) \times p(\xi |\theta_{i} )}}} \right\} $$
  Thus, the updated $\theta_{i + 1}$ is selected as follows:
  4 $$ \theta_{i + 1} = \left\{ {\begin{array}{*{20}l} {\begin{array}{*{20}c} {\theta^{*} } & {if} & {\alpha_{i} \ge u} \\ \end{array} } \\ {\begin{array}{*{20}c} {\theta_{i} } & {\begin{array}{*{20}c} {} & {} \\ \end{array} } & {else} \\ \end{array} } \\ \end{array} } \right. $$
  in which, *u* is a value randomly generated by a uniform distribution that ranges between 0 and 1.
Step III: If the size of the generated sample has yet to reach its threshold (*n*), increase the iteration *i* and repeat *step II;* otherwise, stop.

One should remember that setting the sample size (*n*) requires a trial-and-error process to test whether the *autocorrelation* between the generated values is not significant. Furthermore, given that the process starts at a randomly selected start point there is a possibility that the initial point might be a highly improbable point. Thus, it may take a large number of iterations for the initial position to converge to the posterior distribution. This may be avoided by selecting a suitable initiation point. Readers can refer to Gelman et al.^32^ for more information on the MCMC setting procedures.

One can see the stochastic behavior patterns of the historical data set as the prior distribution in the context of climate change studies, for it describes the pre-existing perception about the probability of observing a given climatic outcome. The generated climate change scenarios via regional climate models (RCMs), in the meantime, can be used to extract the likelihood function. We can interpret these data sets as a plausible sample set of what the future may hold. In this manner, instead of concentrating on a singular sequence of values generated by RCMs, or general circulation models (GCMs) for that matter, the attention would be on the stochastic pattern of behavior of a given climatic variable. Using this setting, thus, one can redefine the pre-excising perception about the impact of climate change by merging what has already happened (i.e., the observed historical data) and a sample of what might happen (i.e., RCMs’ simulations).

The generation of climate change scenarios is herein accomplished by employing the results of one RCM experiment, namely, ICHEC-EC-EARTH from the CORDEX (the coordinated regional climate downscaling experiment) project dataset was implemented in a spatial resolution of 0.22° and at seasonal temporal resolution with respect to climate change scenario RCP 8.5. On an average global scale emission scenario RCP 8.5 would lead to an increased greenhouse effect of 8.3 W/m^2^ in by 2100 on a rising trajectory^33^, meaning that this could potentially have huge impacts on the climate, and in turn, on water resources in many regions. Recent reports, unfortunately, project that these seemingly massive and irreversible impacts of climate change may exceeded the limits that scientists and policymakers previously hoped for^34^. Through this scenario, one could obtain a more pessimistic trajectory for the impacts of climate change, which given the recent developments and findings, seems like a logical path to take. For more information about the used RCM experiment and the climate change scenario the readers can refer to IPCC^14^ and Nikiema et al.^33^. Furthermore, this work employs the Anderson–Darling test to opt for a suitable distribution function. The normal, lognormal, exponential, Weibull, 3-parameter Weibull, extreme value, gamma, logistic, and loglogistic were among the theoretical *pdf*s that were tested for each given data set. Readers are referred to Razali and Wah^35^ for more information about the Anderson–Darling statistical test]. It should be noted that this work focuses on a non-conditional climatic variable (here, air temperature), because for this type of variables, unlike the conditional variables (say, precipitation), it is easier to analyze the stochastic behavior of a phenomenon through a singular *pdf*.

## Case study

The Karkheh River Basin, with a catchment area of 51,000 km^2^, is an iconic basin located in the southwestern region of Iran (latitude: 30–35° N; longitude: 46–49° E). In recent years, decision-makers have searched for a shift toward industrial development plans for the basin, yet the Karkheh River Basin, called the food basket of Iran, remains one of the leading agricultural centers in Iran^36,37^.

**Figure 1:**
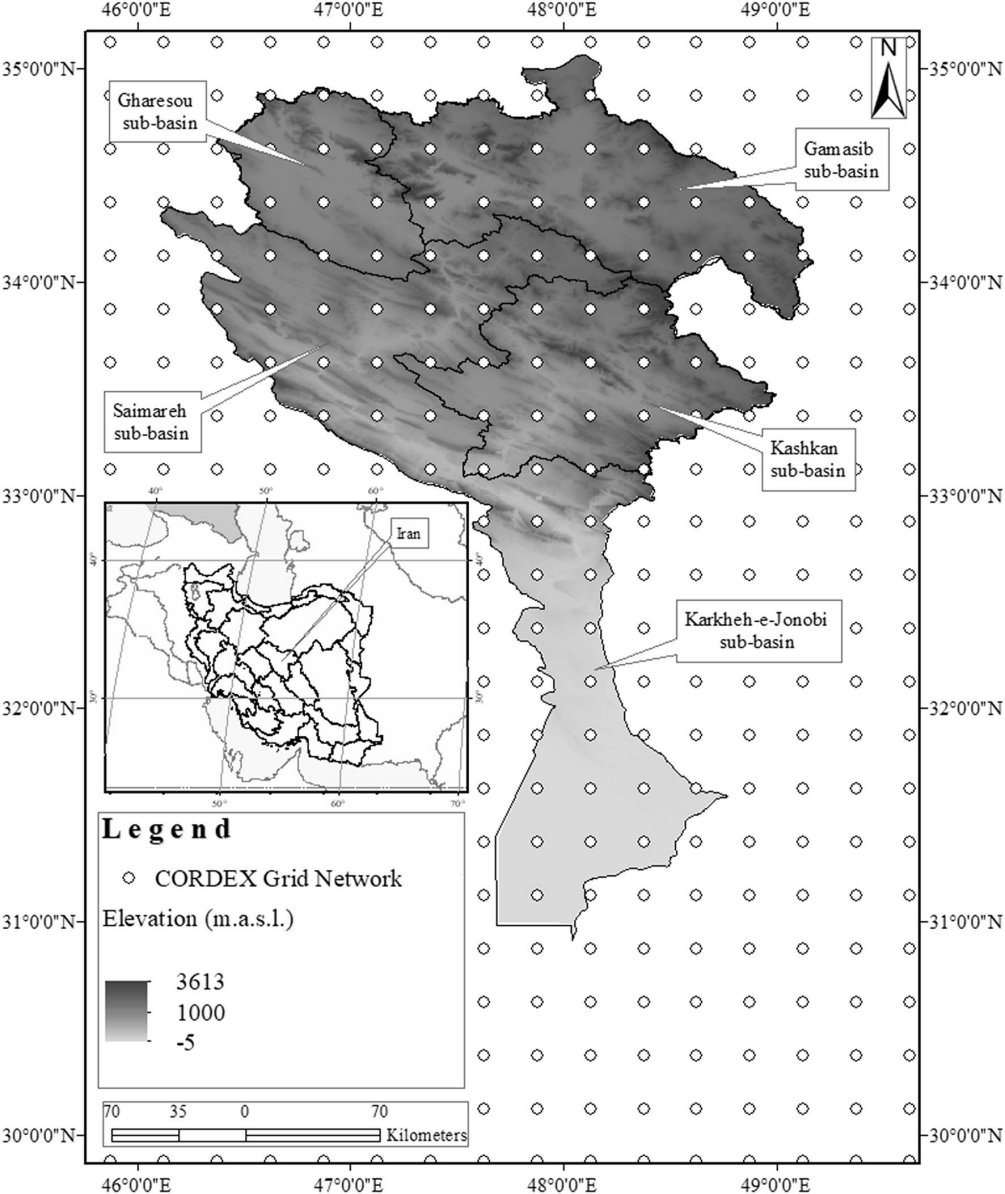
The location of the study area. m.a.s.l. stands for meter above sea level, and CORDEX is short for the coordinated regional climate downscaling experiment.

The Karkheh River Basin is composed of five sub-basins, namely, Gamasiab, Gharesou, Karkheh-e-Jonobi, Kashkan, and Saimareh (Fig. 1). Two-thirds of the basin has an elevation higher than 3500 m above sea level (m.a.s.l.), which mostly describe the north and northeast of the basin. On the other hand, one-third of the basin’s elevation is lower than 10 m.a.s.l., which is primarily the case for the southern part of the basin.

**Figure 2:**
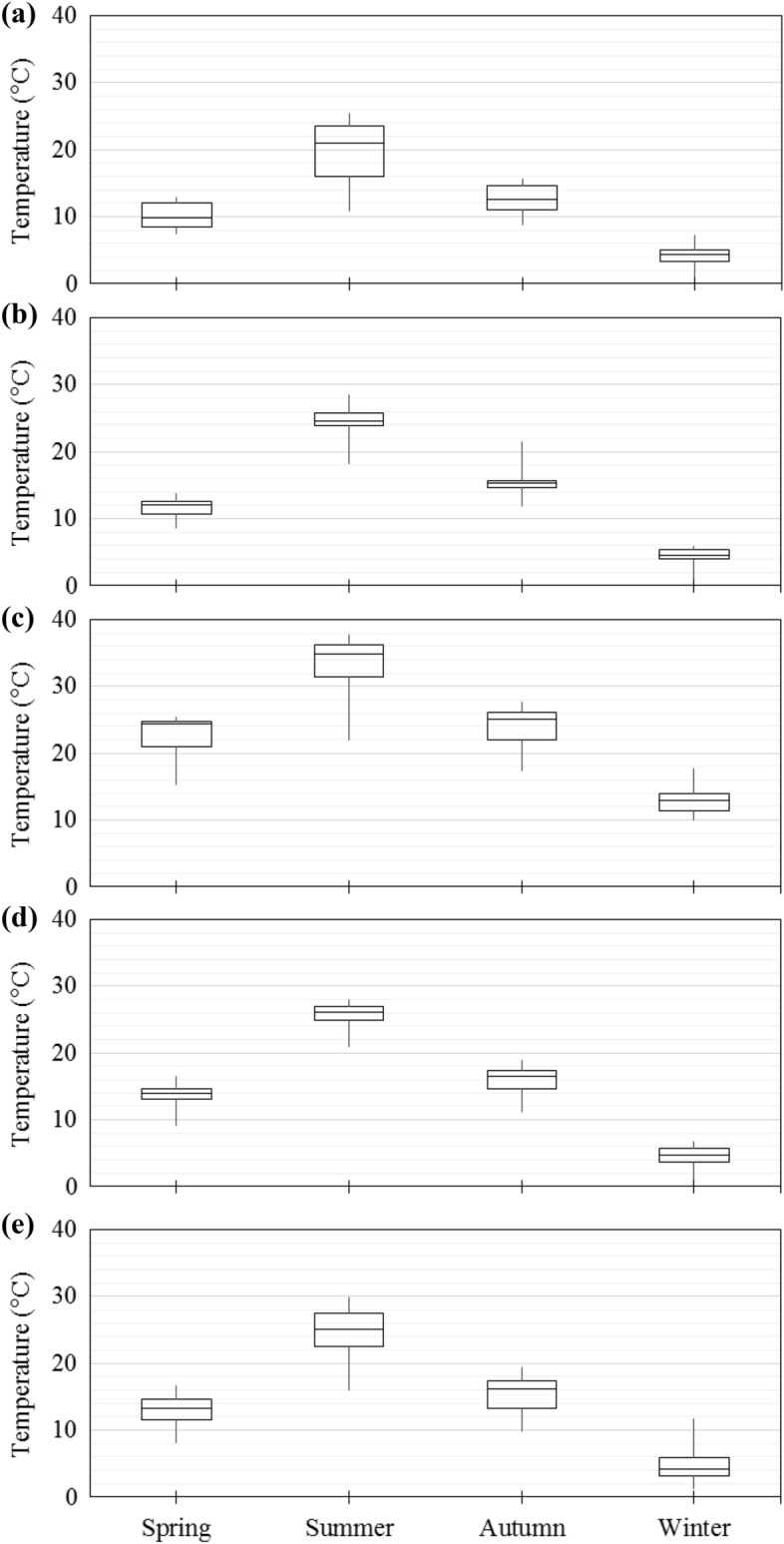
Average monthly variation of temperature in different sub-basins of the Karkheh River Basin in the baseline period (1975–2005): **(a) **Gamasiab; **(b)** Gharesou; **(c)** Karkh-e-Jonobi; **(d)** Kashkan; and **(e)** Seimareh.

The Karkheh River Basin is mainly characterized as an arid to semi-arid region. The basin is composed of three main classes based on the Köppen − Geiger climate classification. The northern, mid, and southern sections of the basin are classified as Dsa, Csa, and Cwa, respectively (D: snow; C: warm temperature; s: summer dry; w: winter dry; a: hot summer). See Kottek et al.^38^, Rubel & Kottek^39^ and Rubel et al.^40^ for more information on the Köppen − Geiger climate classification system. The basin also experiences a high level of spatiotemporal variation when it comes to rainfall and air temperature variables^41^. The southern part of the basin (e.g., Karkheh-e-Jonobi sub-basin) usually experiences hotter weather in comparison to the northern parts (e.g., Gamasiab sub-basin). Figure 2 illustrates the average monthly variation temperature in different sub-basins of the Karkheh River Basin in the baseline period (1975–2005).

The reasons for selecting this case study were twofold. The first reason is rooted in the critical role of the Karkheh River Basin in Iran’s water and food security. The Karkheh River Basin is a major source of water, food, and now energy, thanks to the booming era of hydropower development in the region^42^. Furthermore, recent studies suggest that climate change has started to make an impact on the climatic status of the Middle East and North African (MENA) nations^43^. These changes in the climate must be studied and understood to make informed decisions and devise the proper adaptation plans. A central theme of this study is to address the deep uncertainties that are associated with the climate-change phenomenon. The second reason is that the Karkheh River Basin is a topographically diverse region. It is a known fact that in this type of situation the projections of GCMs made with coarse numerical grids are downscaled to finer-scale numerical grids. RCMs, for instance, is one of the options available to downscale the GCM projections to obtain representative projections of the regions’ spatial variations. This study employs the CORDEX datasets (ICHEC-EC-EARTH model; and RCP 8.5 scenario) to project the temperature variable averaged over three months under climate change conditions. The said values represent the corresponding seasonal average changes in spring, summer, autumn, and winter.

## Results and discussion

As stated above this study aims to shed light on the deep uncertainties that are associated with the climate change phenomenon. The seasonally-averaged surface air temperature, hereafter simply referred to as temperature, was selected as the non-conditional climatic variable to be monitored within the Karkheh River Basin, Iran, during the baseline period (1975–2005). The CORDEX datasets (RCP 8.5) were employed to make climate-change projections.

The first step in the proposed framework is to identify the most suitable theoretical distribution function to represent the stochastic behavior patterns of both historical and climate change data sets. Such identification considered the following theoretical distributions: normal, lognormal, exponential, Weibull, 3-parameter Weibull, extreme value, gamma, logistic, and loglogistic. It is important to note here that the primary strategy in this study is to analyze the data from a numeric standpoint without any presumption about the stochastic structure of the data^44^. As such, the study would opt for any distribution that is deemed fittest to describe the data. A summary of the fitted distributions to represent the prior distributions and likelihood functions is found in Tables S1 through S4 (see the Appendix). Furthermore, the climate-change period was divided into three mutually exclusive time frames which are short-term (2010–2039), mid-term (2040–2069), and long-term (2070–2099) future to gain a better understanding of the evoluton of future temperature changes.

**Figure 3:**
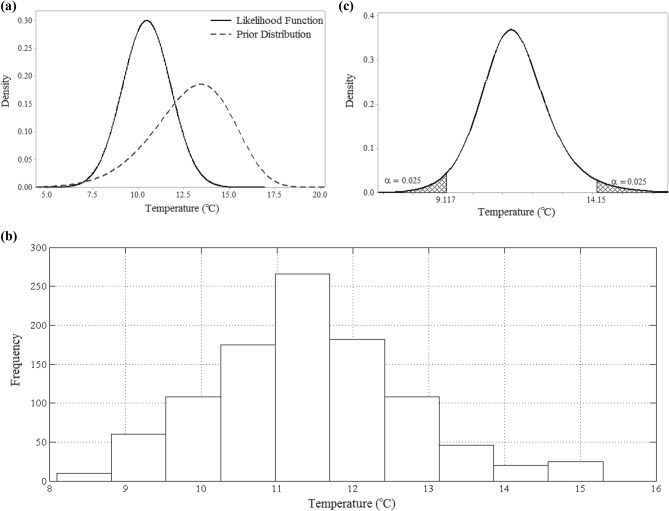
The step-by-step process of computing the posterior *pdf*: **(a)** the prior distribution of Seimareh sub-basin during spring and the likelihood function of this sub-basin in the *short*-*term* future; **(b)** the histogram of the generated samples; and **(c)** the posterior distribution.

With Bayes’ theorem in mind, a Markov Chain Monte Carlo (MCMC) method was then applied to merge the prior distributions and the likelihood functions and to generate a sample set from the posterior distribution set. After a series of trials-and-errors, the sample size for the MCMC algorithm was set to be 1000 (*n* = 1000). These generated sample sets were then used to specify the most suitable theoretical *pdf*s to represent the posterior distribution functions. Figure 3, for instance, illustrates the most appropriate theoretical distribution that could represent the posterior distribution for the Seimareh sub-basin during spring under the short-term period.

**Figure 4:**
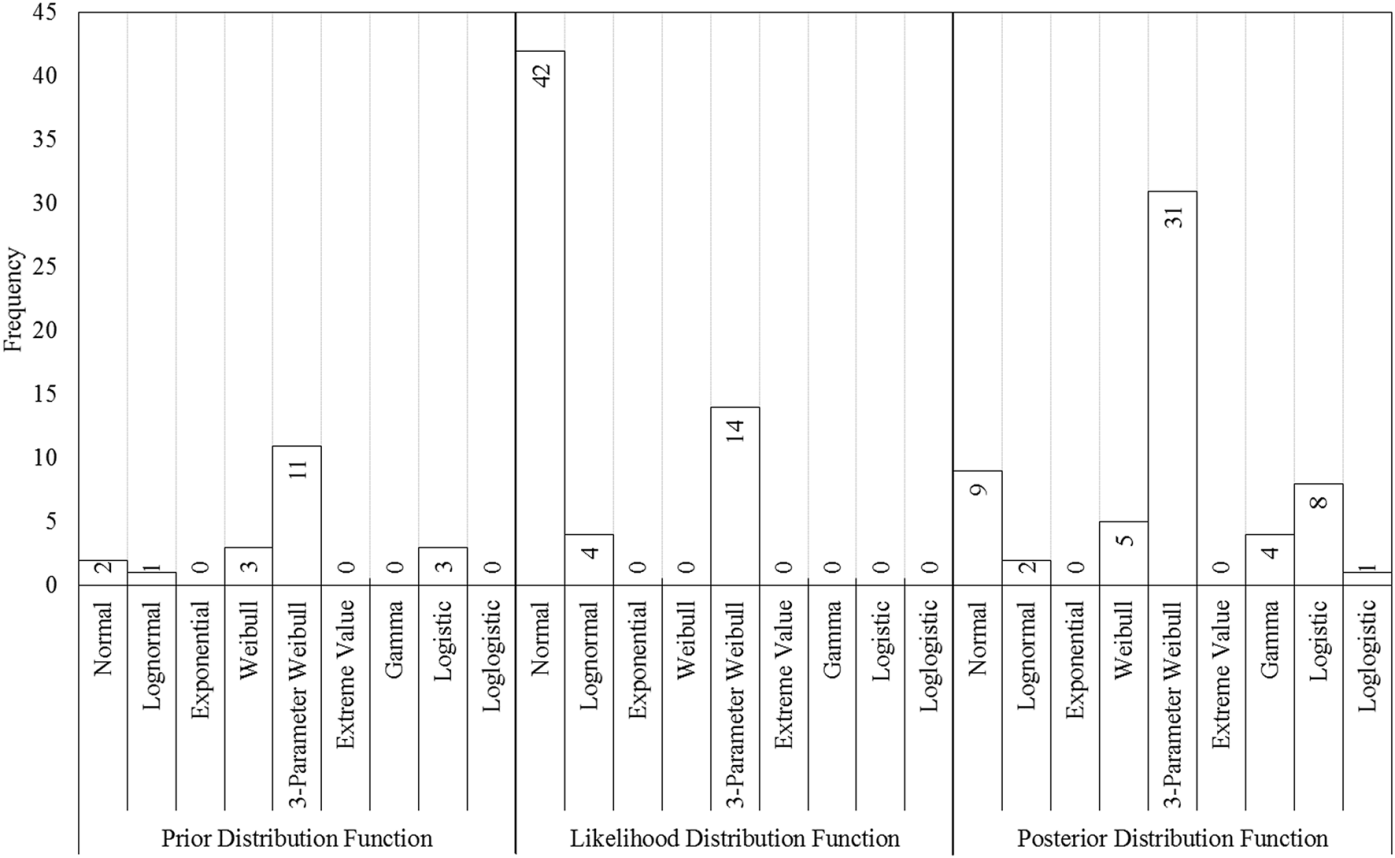
The frequency of using each individual theoretical *pdf*s as prior, likelihood function, and posterior distributions.

Figure 4 demonstrates the frequency with which each specific theoretical distribution functions was deemed the most suitable to characterize the prior, likelihood, and posterior distributions. Analyzing the fitted *pdf*s in Fig. 4 reveals an important point about the nature of RCMs’ raw projections. Specifically, the most frequently chosen distribution function for prior and posterior distributions is the 3-parameter Weibull. As for the likelihood function, however, it was the normal distribution that outperformed other available alternatives. Furthermore, the type of selected theoretical distribution for prior and posterior *pdf*s seems far more diverse compared to those from the likelihood functions. In fact, the likelihood functions were only limited to three types of distributions, most of which are normal distributions. Keep in mind that these functions are the most suitable *pdf*s that were fitted to the RCMs projected results. The cause behind this notion might be traced back to the nature of RCMs’ projections. RCMs operate at a finer horizontal resolution than GCMs, and thus they provide localized and high-resolution detailed climatic information that can be of importance for many management purposes, especially in regions with complex topography. However, the analyzed data revealed that among the distributions fitted for the likelihood function the normal distribution was found to be the best distribution to describe the data 70% of the time. This could be interpreted as signaling that employing RCMs’ raw projections, especially for regions that have considerable volatility in their climatic variables, should be used with caution, and further adjustment to the raw projected data may be required in some cases. Note that from a statistical standpoint, the normal distribution is not heavy-tailed, and as such, may not be the best way to portray this data. The fact that, in most cases, it has been selected as the best way to portray the stochastic nature of the likelihood function (i.e., RCM’s projections) means that innate characteristics of these data might prevent them to truly represent these types of variables on their own.

**Figure 5:**
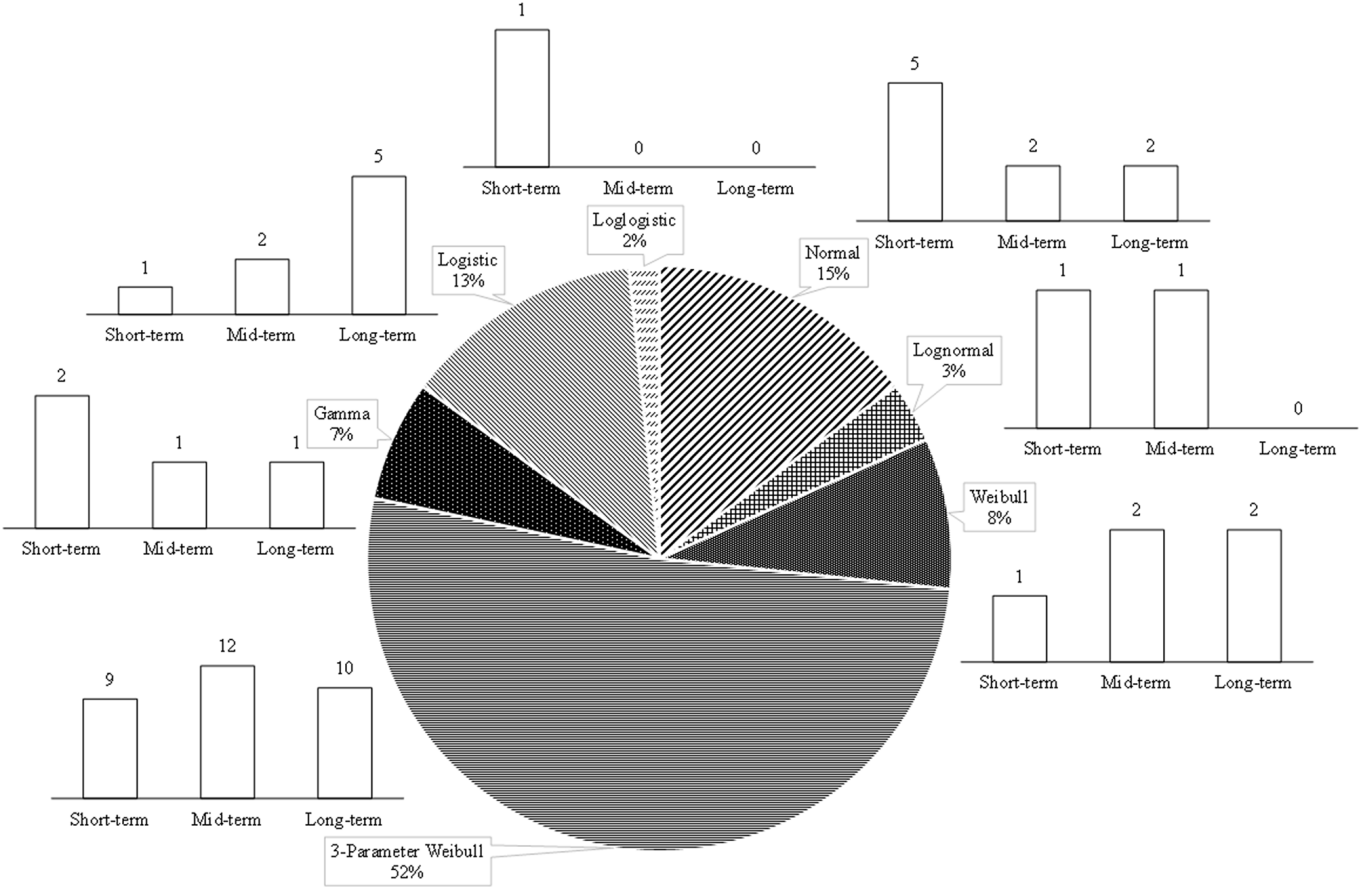
The frequency of using different theoretical distributions as posterior *pdf*s.

Figure 5 provides additional information regarding the frequency in which each individual theoretical distributions were deemed suitable to represent the posterior *pdf*s. While posterior distribution sets are, indeed, the most diverse in terms of the number of different types of distributions, a significant proportion of fitted *pdf*s (approximately 52%), however, are fitted by the 3-parameter Weibull distribution. Further information regarding the fitted distributions to represent the posterior *pdf*s is found in Tables S5 to S7 (Appendix).

**Figure 6:**
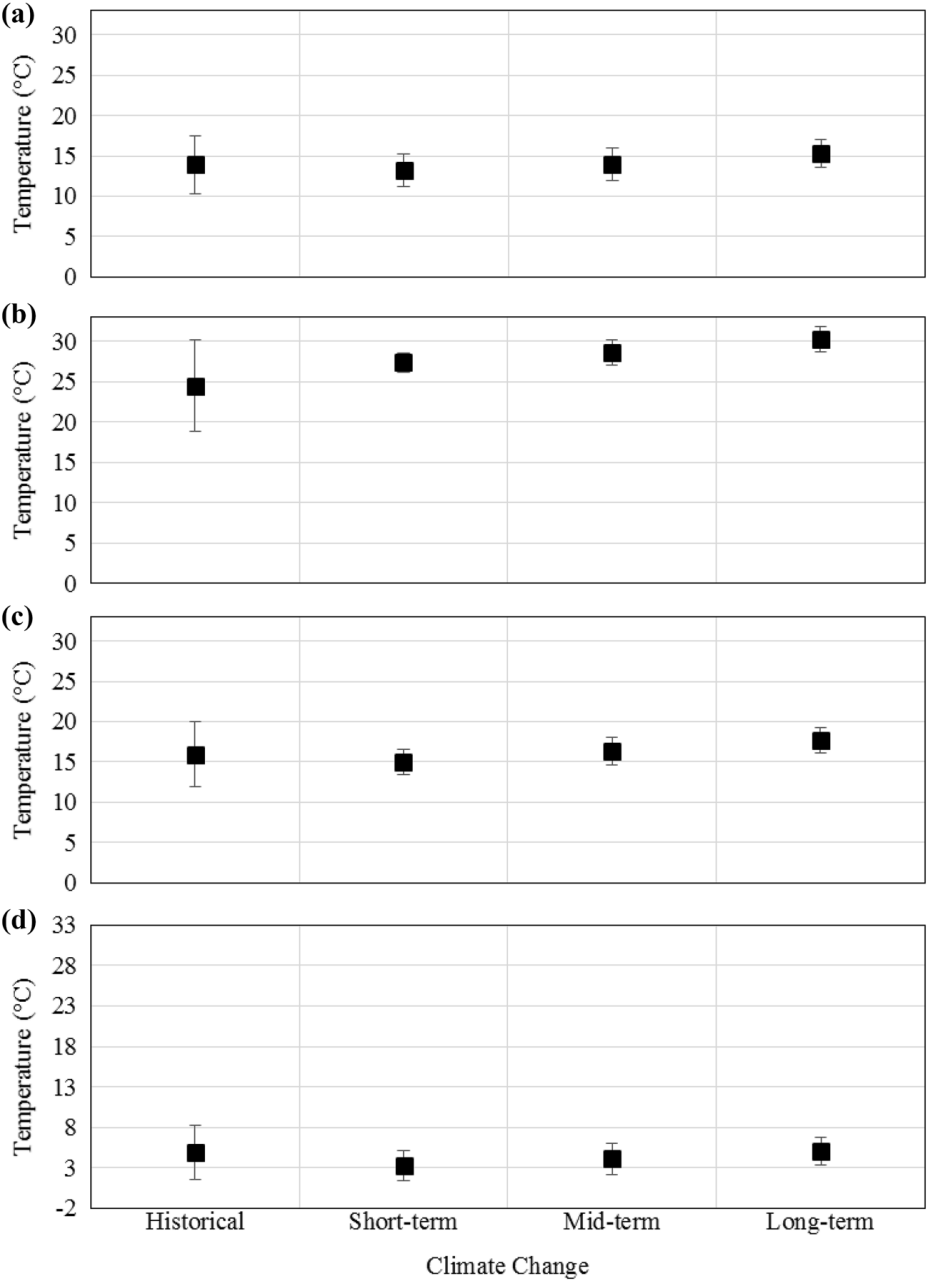
The historical and simulated average temperatures of the entire basin with the 95%confidence interval in **(a)** spring; **(b)** summer; **(c)** autumn; and **(d)** winter.

The computed posterior distribution functions can be interpreted as modified representations of the stochastic behavior of temperature variable concerning the short-term, mid-term, and long-term climate change projections. In that spirit, employing the confidence interval of 95%, the average temperature of the entire basin is depicted in Fig. 6 associated with historical and climate change conditions. Two sets of behavior patterns are observed. The first one is a broad trend in summer. The second pattern describes the rest of the seasons. In summer (Fig. 6b) the presence of a mild, yet, steady positive trend (upward) is detected. Here, one can expect the average temperature of the basin to increase steadily with the passage of time. As for the rest of the seasons, while it seems that the average temperature of the basin would experience a mild drop in the short-term, the temperature would begin to rise with a steady trend with time. In spring (Fig. 6a) and autumn (Fig. 6c) time series, it is projected that the expected average temperature in the basin would eventually surpass those that had been experienced in the baseline condition in the mid-term and long-term future. Concerning winter temperature it is seen in Fig. 6d that it is projected to increase over time. Yet, it has been estimated that it might not reach the observed average temperature of the basin in neither of the expressed time frames. Of course, given the upward trend in the data, this temperature would indeed be reached in a longer timeframe. It is worth noting that these patterns are in line with the idea that the earlier impacts of climate change are to amplify the historical patterns in climatic variables. That is why the data show a slight drop in colder seasons and an uptick in the warmer ones. That is, of course, until eventually, a new climatic equilibrium is reached on a global scale. At this point, the temperature as shown here would start to increase gradually.

The other notable implication that can be understood by analyzing Fig. 6 is the variation in the width of the confidence intervals under baseline and climate change conditions. In comparison to the baseline condition, the length of the 95% confidence intervals would dramatically decrease under climate change conditions. This shrinking indicates that the generated results are more densely surrounding the central tendency measure herein chosen as the mean (*μ*) of the data. This notion is still in line with the idea that RCM’s projections mostly resemble the stochastic characteristic of normal distributions. A normal distribution is by nature not a heavily-tailed distribution, meaning that it rarely generates tail values. Even though the MCMC framework has mitigated this effect to some extent, they inevitably inherit this stochastic property from the likelihood functions.

Again, to truly understand the obtained results here, one must first acknowledge how Bayesian models work. The main idea behind a Bayesian-based framework is to adjust the prior assumptions about a stochastic phenomenon through observed samples. In this case, the prior information represents the historical data, and the likelihood function (i.e., the samples) is obtained from RCM projects. As can be seen here, while RCMs’ projections might be perfectly capable of portraying the normal behavior of a variable under climate change conditions, which is usually sufficient for most lumped evaluation of climate change impact assessments, they might not be suitable to study extreme hydro-climatic events. The main problem with the raw RCM projections is that they follow a normal distribution, which is a symmetric distribution. Figure 4 suggests that while the MCMC framework here is mitigating this impact the final projections inherit this property from the likelihood functions. This simply means that while any RCM-based projection is perfectly suitable to understand the general outline of the climate change impacts, they are not the best option to study extreme events because even by modifying their *pdf*s, they rarely generate truly extreme values. The average temperatures in all sub-basins under baseline and climate change conditions are summarized in Table 1.

**Table 1:** The average surface air temperature in all sub-basins under baseline and climate change conditions (°C).

Sub-basin	Baseline				Short-term				Mid-term				Long-term			
	Spring	Summer	Autumn	Winter	Spring	Summer	Autumn	Winter	Spring	Summer	Autumn	Winter	Spring	Summer	Autumn	Winter
Gamasiab	10.8 ± 3.8	18.0 ± 8.1	12.7 ± 4.4	3.0 ± 3.1	8.6 ± 1.6	23.1 ± 1.2	10.8 ± 1.5	2.2 ± 1.4	9.5 ± 1.9	24.6 ± 1.7	12.2 ± 1.9	2.7 ± 1.9	10.9 ± 1.9	26.5 ± 1.7	13.5 ± 1.6	2.4 ± 1.3
Gharesou	11.4 ± 2.4	24.7 ± 3.9	15.1 ± 1.4	2.4 ± 2.2	10.8 ± 1.7	25.1 ± 1.4	13.0 ± 1.5	1.0 ± 2.6	11.8 ± 1.8	26.4 ± 1.7	14.4 ± 1.5	1.6 ± 2.1	12.7 ± 1.4	28.4 ± 2.1	15.4 ± 1.6	2.7 ± 1.6
Kharkh-e-Jonobi	21.7 ± 4.7	31.4 ± 7.3	22.7 ± 5.4	12.9 ± 3.6	21.8 ± 1.9	35.8 ± 1.1	22.8 ± 1.7	9.6 ± 2.0	22.7 ± 2.3	37.4 ± 1.6	24.4 ± 2.0	11.0 ± 2.0	24.4 ± 1.9	39.1 ± 1.6	25.9 ± 1.6	11.8 ± 1.9
Kashkan	13.3 ± 2.8	25.0 ± 3.0	15.0 ± 3.6	3.4 ± 3.5	13.0 ± 2.1	27.2 ± 0.9	15.1 ± 1.6	1.9 ± 2.1	13.9 ± 1.7	27.6 ± 1.1	16.3 ± 1.5	2.7 ± 1.6	14.7 ± 1.5	28.5 ± 0.8	17.4 ± 1.5	4.7 ± 1.7
Seimareh	12.4 ± 4.3	23.3 ± 6.0	14.1 ± 5.4	5.0 ± 4.1	11.7 ± 2.7	25.7 ± 1.2	13.1 ± 1.5	1.4 ± 1.2	12.1 ± 2.3	27.1 ± 1.7	14.5 ± 1.8	2.5 ± 2.0	13.9 ± 2.0	29.1 ± 1.8	16.4 ± 1.6	3.4 ± 2.2

The recorded values are within a 95% confidence interval.

As for the impact of climate change, it is clear that these data are associated with deep uncertainty; that is, the parameters used to describe the stochastic behavior of a variable may be subjected to some degree of uncertainty. These parameters, *μ* for one, may also be represented by a *pdf* of their own. This study focuses on highlighting this type of deep uncertainty that might interfere with the central tendency measure *μ*.

The deep uncertainty in this instance dictates that the recorded parameters for each posterior distribution are not deterministic values. While for a given prior distribution and likelihood function the MCMC would lead to a specific type of posterior *pdf*, the parameters that are used to define this *pdf* (e.g., *μ*), could vary each time the algorithm is used. If this variation is mild, there is more certainty about the nature of the variable’s stochastic behavior pattern (i.e., the posterior distribution function). If it is determined that the parameters are experiencing severe variations then the deep uncertain environment would leave the decision-makers unsure about the variables’ stochastic behavior pattern.

**Figure 7:**
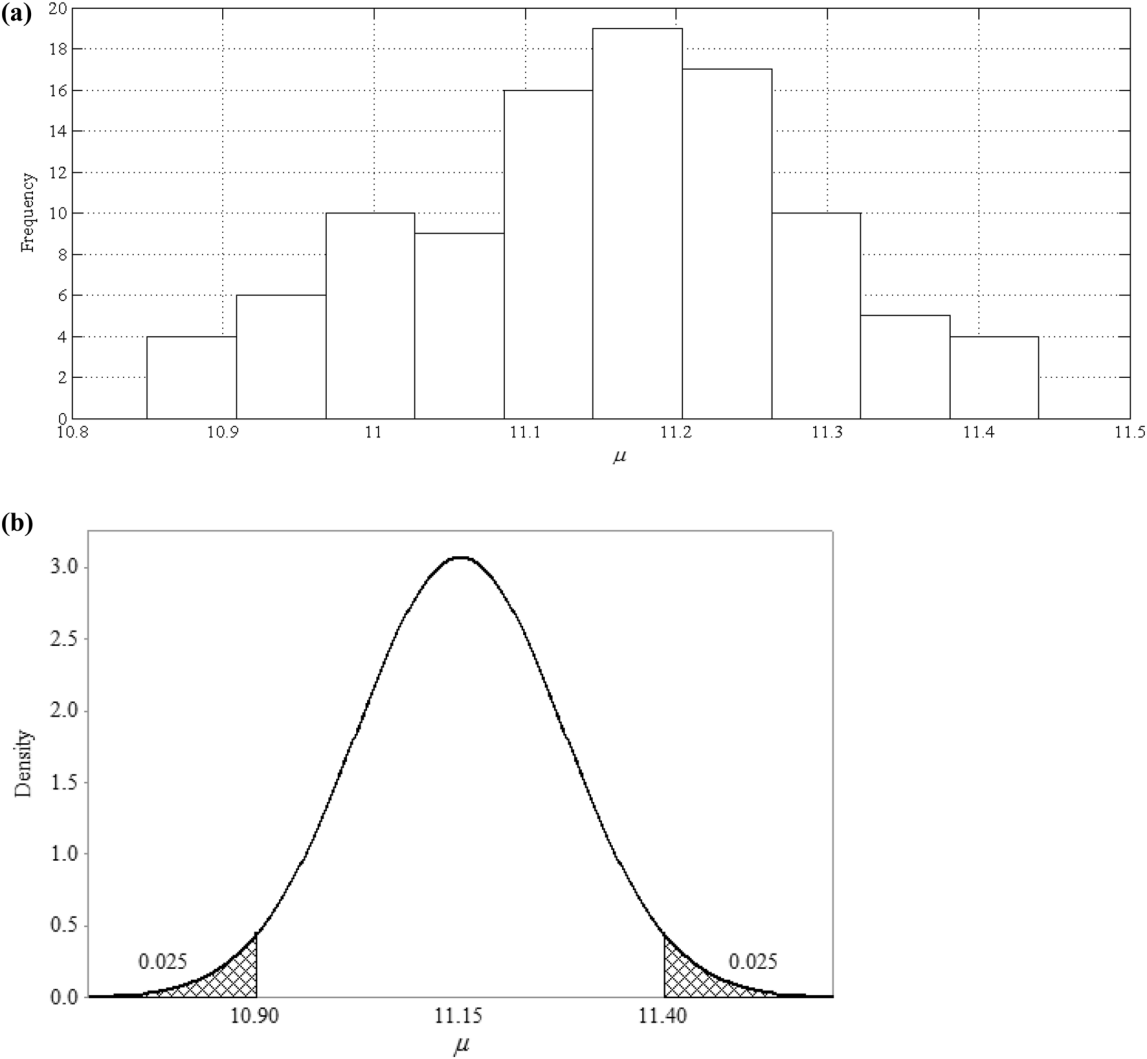
The uncertainty of the computed *μ* parameter for Seimareh sub-basin in spring under short-term future condition demonstrated by **(a)** a histogram and **(b)** a probability distribution function.

With that idea in mind the combination of prior distributions and likelihood functions was executed for 100 times, and in each iteration the mean of 1000 samples was recorded. A theoretical distribution function was then fitted to the recorded values. Naturally, if the recorded values are generally close to one another numerically, the parameters of the computed posterior *pdf*s are less subjected to deep uncertainty. If, however, these values show significant fluctuation then the deep uncertainty of climate change would impede predictions of the stochastic behavior pattern of temperature. Figure 7, for example, portrays the uncertainty of the computed *μ* parameter for Seimareh sub-basin in spring under short-term future condition.

**Figure 8:**
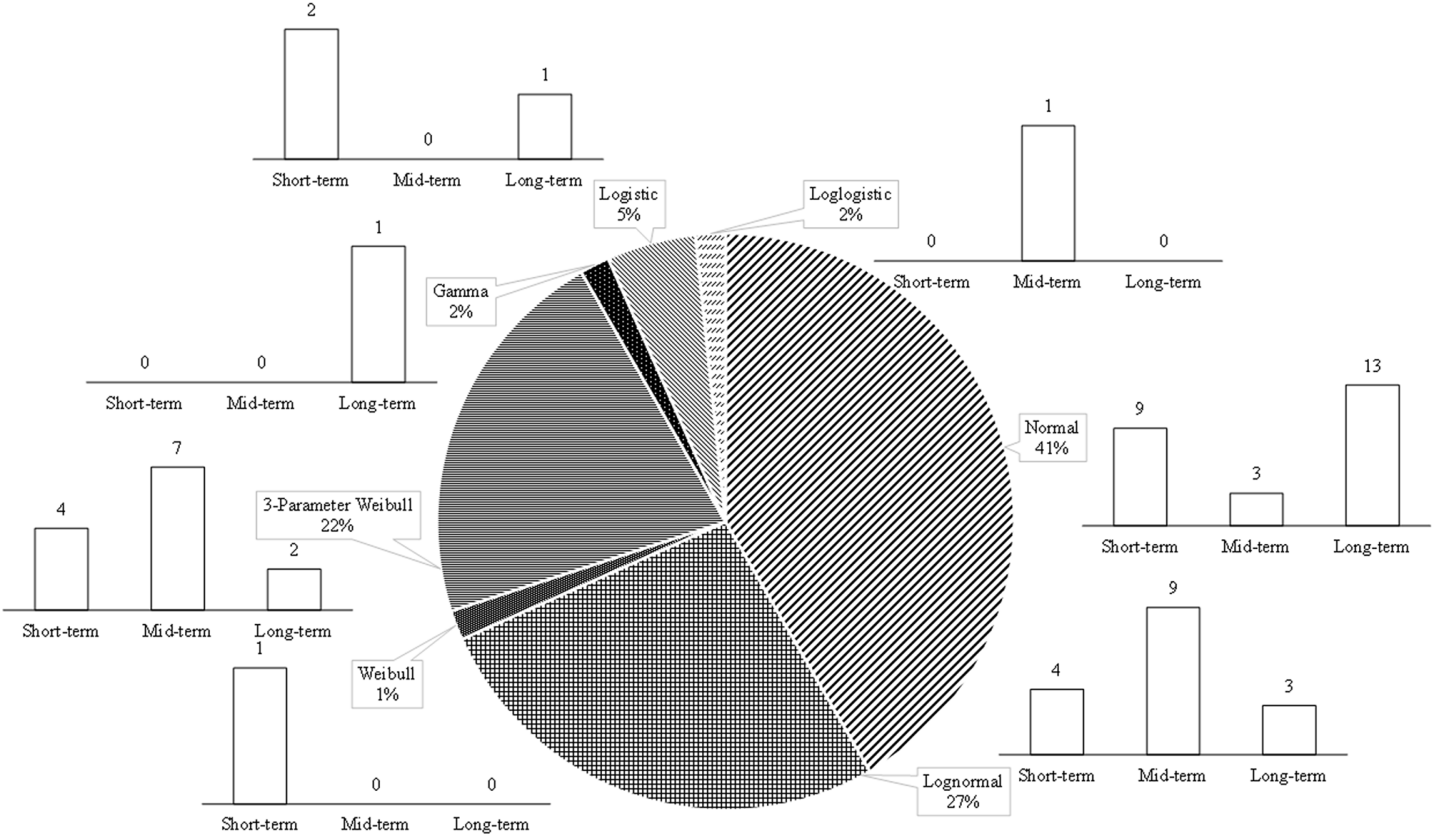
The frequency with which each theoretical distribution was found suitable to describe the stochastic distribution of the *μ* parameter.

Figure 8 demonstrates the number of times each theoretical distribution was chosen to portray the stochastic behavior of the *μ* parameter. As can be seen here, the normal and lognormal distributions are the most common *pdf*s used to describe the variation in the *μ* parameter. One should also note the fact that about 65% of the distributions used to describe the future condition are normal distributions. The list of fitted *pdf*s is summarized in Tables S8 to S10.

Table 2 summarizes the variation in the computed *μ* parameter in each given sub-basin. It is seen in Table 2 the 95% confidence interval of the *μ* parameter in all cases ranges between ± 0.1 and ± 0.3 °C. In 55% of the cases, this interval was found not to be more than ± 0.1 °C, and, furthermore, in 97% of them the interval was less than ± 0.2 °C. Needless to say, a widened confidence interval for the *μ* parameter can only signal that the deep uncertainty has a more pronounced impact on the temperature’s stochastic behavior. As for the case of the spring data set of the Seimareh sub-basin under the short-term condition, or the case of the Gharesou sub-basin’s winter data series under short-term period, the confidence interval for the *μ* parameter is estimated to be ± 0.3 °C wide. This indicates that compared to other projected posterior *pdf*s there is less certainty about the predicted stochastic behavior pattern of temperature variable for these particular cases. As shown in Table 2 in some cases, the variation in the projected *μ* temperature’ posterior *pdf*s is decreasing over time (for a given season over different timeframes). As discussed earlier, this was interpreted as the deep uncertainties of the climate change projections, meaning that lower volatility in this measure indicates that the said variable is less affected by the deep uncertainty of the climate-change phenomenon. This observation is in line with the general belief that, in the near future, the climate change phenomenon is most likely to intensify the historical patterns in climatic variable, but gradually we expect to see an upward trend in temperature in the longer run^45^. In this case, there is more volatility in the earlier time frames, but as time progresses, this volatility seems to decrease in some cases. This means that the obtained projections are showing less uncertainty about the outline behavior of the parameter for the long-term future as the models that are simulating the climatic behavior under climate change conditions have already reached a new equilibrium by that point.

**Table 2:** The variation in the computed *μ* of the temperature’ posterior *pdf*s.

Sub-Basin	Short-term				Mid-term				Long-term			
	Spring	Summer	Autumn	Winter	Spring	Summer	Autumn	Winter	Spring	Summer	Autumn	Winter
Gamasiab	8.6 ± 0.2	23.1 ± 0.1	10.7 ± 0.1	2.0 ± 0.1	9.6 ± 0.2	24.6 ± 0.1	12.1 ± 0.2	2.5 ± 0.2	11.0 ± 0.2	26.5 ± 0.1	13.6 ± 0.2	2.4 ± 0.1
Gharesou	10.8 ± 0.1	25.1 ± 0.1	13.2 ± 0.1	1.0 ± 0.3	12.0 ± 0.1	26.3 ± 0.1	14.4 ± 0.1	1.9 ± 0.2	12.5 ± 0.1	28.4 ± 0.2	15.4 ± 0.1	3.0 ± 0.1
Kharkh-e-Jonobi	22.1 ± 0.2	35.9 ± 0.1	22.9 ± 0.2	9.6 ± 0.2	23.2 ± 0.2	37.3 ± 0.1	24.4 ± 0.2	10.7 ± 0.2	24.4 ± 0.2	39.0 ± 0.1	26.0 ± 0.1	12.0 ± 0.2
Kashkan	13.1 ± 0.2	27.3 ± 0.1	15.2 ± 0.1	1.9 ± 0.2	14.0 ± 0.2	27.8 ± 0.1	16.3 ± 0.1	2.8 ± 0.1	14.8 ± 0.1	28.5 ± 0.1	17.6 ± 0.1	4.8 ± 0.2
Seimareh	11.2 ± 0.3	25.7 ± 0.1	13.2 ± 0.1	1.3 ± 0.1	12.4 ± 0.2	27.3 ± 0.1	14.7 ± 0.2	2.2 ± 0.2	13.8 ± 0.2	29.1 ± 0.1	16.4 ± 0.1	3.4 ± 0.2

The recorded values are within a 95% confidence interval.

## Concluding remarks

Deep uncertainties of climate change need to be understood and accounted for when climatic variables are of concern. Broadly speaking, this level of uncertainty refers to a situation where the parameters used to define the probability distributions of a stochastic phenomenon are themselves subject to some degree of uncertainty. This study aimed to shed light on this matter by employing an MCMC-oriented framework. According to the Bayesian theorem, the previous knowledge about a stochastic phenomenon (i.e., prior distribution) can be modified using the additional information gathered from a representative sample (i.e., likelihood function). The MCMC simulations provide a framework to carry such computations and estimate the adjusted stochastic behavior pattern of a given phenomenon (i.e., posterior distribution). To demonstrate this notion, we selected Karkheh River Basin, Iran, as a case study. The historical datasets were fitted with nine *pdf*s, which were to serve as the prior distributions. As for the likelihood functions, the CORDEX datasets were applied to project the temperature under climate change conditions. The generated values were then fitted with a series of *pdf*s to represent the likelihood functions. We then employed the MCMC algorithm to extract the posterior distributions.

Analyzing the data with a seasonally time step revealed two sets of behavior patterns for the climate change data sets. In summer, a mild, yet, steady positive trend was detected. Thus the evidence suggests that as time goes on one can expect the average temperature of the basin to increase steadily during summer times. As for the rest of the seasons it seems that the average temperature of the basin would experience a mild drop in the short-term, but the temperature would begin to rise with a steady trend with time. In the spring and summer this increase in temperature would eventually lead to an average temperature higher than what has already been experienced in the baseline period. In winter, however, while the temperature is projected to increase over time it has been estimated that it might not reach the observed average temperature of the basin in the foreseeable future.

This study also aimed to shed light on the deep uncertainty associated with climate change phenomenon. In that regard, each posterior distribution was regenerated, and their measure of central tendency (*μ*) was recorded. A series of *pdf*s were then fitted to these gathered data sets. The 95% confidence interval of these *pdf*s ranged between 0.1 and 0.3 °C. It was also revealed that as time progresses, the *μ* parameter is less affected by the deep uncertainty of the climate change phenomenon. As a side note, analyzing the likelihood functions suggested that employing RCMs’ raw projections in a topographically diverse region should be proceed with caution because they rarely generate truly extreme values. The results of this study suggest that RCMs’ protections often resemble the stochastic properties of non-heavily-tailed distributions. As such, while these results could be perfectly suitable to understand the general impact of climate change on a regional scale, they may not be able to reveal much about the impact of extreme hydro-climatic variables.

## Supplementary Information

Supplementary Tables.

## Data Availability

The data that support the findings of this study are available from the corresponding author upon reasonable request.
