# Supplementary material for: Sensitivity of non-conditional climatic variables to climate-change deep uncertainty using Markov Chain Monte Carlo simulation
Source: Sci Rep. 2022 Feb 2;12:1813. doi: 10.1038/s41598-022-05643-8 (PMC8810780; doi:10.1038/s41598-022-05643-8)
Supplement: Supplementary file 1 — Supplementary Tables. [file 41598_2022_5643_MOESM1_ESM.doc]

**Appendix**:

**Table S1.** The selected parameters of the fitted prior distribution functions.

| Station | Season | Distribution | Parameters’ Description |
| --- | --- | --- | --- |
| Gamasiab | Spring | Lognormal | μ (Location): 2.30472; σ (Scale): 0.19038 |
|  | Summer | 3-Parameter Weibull | Scale: 5560.43535; Shape: 1703.06355; Threshold: -5538.61826 |
|  | Autumn | Normal | μ (Location): 12.64668; σ (Scale): 2.24461 |
|  | Winter | Logistic | μ (Location): 3.00324; σ (Scale): 0.84140 |
| Gharesou | Spring | Weibull | Scale: 12.31482; Shape: 11.65845 |
|  | Summer | Logistic | μ (Location): 24.72037; σ (Scale): 1.05647 |
|  | Autumn | Logistic | μ (Location): 15.05993; σ (Scale): 0.71544 |
|  | Winter | 3-Parameter Weibull | Scale: 590.90683; Shape: 673.10952; Threshold: -587.50318 |
| Karkheh-e-Jonobi | Spring | 3-Parameter Weibull | Scale: 8577.70902; Shape: 4533.72437; Threshold: -8553.80545 |
|  | Summer | 3-Parameter Weibull | Scale: 11578.19618; Shape: 3929.03961; Threshold: -1.15433E+04 |
|  | Autumn | 3-Parameter Weibull | Scale: 9771.85718; Shape: 4515.92084; Threshold: -9746.55283 |
|  | Winter | Normal | μ (Location): 12.88040; σ (Scale): 1.84895 |
| Kashkan | Spring | 3-Parameter Weibull | Scale: 13.24389; Shape: 10.60828; Threshold: 1.08614 |
|  | Summer | 3-Parameter Weibull | Scale: 12125.77026; Shape: 10202.30382; Threshold: -1.20994E+04 |
|  | Autumn | 3-Parameter Weibull | Scale: 5729.25782; Shape: 3967.55403; Threshold: -5712.56127 |
|  | Winter | 3-Parameter Weibull | Scale: 521.99890; Shape: 370.65769; Threshold: -516.96598 |
| Seimareh | Spring | Weibull | Scale: 13.78224; Shape: 6.86020 |
|  | Summer | 3-Parameter Weibull | Scale: 9008.93455; Shape: 3370.72197; Threshold: -8982.80721 |
|  | Autumn | 3-Parameter Weibull | Scale: 5084.09102; Shape: 2347.95666; Threshold: -5067.40828 |
|  | Winter | Weibull | Scale: 5.06801; Shape: 2.19196 |

**Table S2 |** The selected parameters for the fitted likelihood distribution functions in the short-term future (2010-2039).

| Station | Season | Distribution | Parameters’ Description |
| --- | --- | --- | --- |
| Gamasiab | Spring | 3-Parameter Weibull | Scale: 2025.12294; Shape: 1509.77040; Threshold: -2016.92378 |
|  | Summer | Normal | μ (Location): 23.18811; σ (Scale): 0.62078 |
|  | Autumn | Normal | μ (Location): 10.41320; σ (Scale): 0.80879 |
|  | Winter | Normal | μ (Location): -5.13328; σ (Scale): 2.08419 |
| Gharesou | Spring | 3-Parameter Weibull | Scale: 11.16178; Shape: 9.70415; Threshold: -0.75934 |
|  | Summer | Normal | μ (Location): 25.15935; σ (Scale): 0.67633 |
|  | Autumn | Normal | μ (Location): 12.37870; σ (Scale): 0.86056 |
|  | Winter | Normal | μ (Location): -2.07204; σ (Scale): 1.85984 |
| Karkheh-e-Jonobi | Spring | 3-Parameter Weibull | Scale: 8.92427; Shape: 8.27450; Threshold: 13.26446 |
|  | Summer | 3-Parameter Weibull | Scale: 4.00454; Shape: 8.11309; Threshold: 32.22300 |
|  | Autumn | Normal | μ (Location): 22.72187; σ (Scale): 0.80927 |
|  | Winter | Lognormal | μ (Location): 2.11752; σ (Scale): 0.10774 |
| Kashkan | Spring | Normal | μ (Location): 12.45076; σ (Scale): 1.33800 |
|  | Summer | Normal | μ (Location): 27.66977; σ (Scale): 0.60156 |
|  | Autumn | Normal | μ (Location): 14.85585; σ (Scale): 0.85394 |
|  | Winter | Normal | μ (Location): 1.15035; σ (Scale): 1.12084 |
| Seimareh | Spring | Normal | μ (Location): 10.48784; σ (Scale): 1.33472 |
|  | Summer | Normal | μ (Location): 25.72986; σ (Scale): 0.63359 |
|  | Autumn | Normal | μ (Location): 12.97521; σ (Scale): 0.82401 |
|  | Winter | 3-Parameter Weibull | Scale: 8.22753; Shape: 6.22099; Threshold: -8.60928 |

**Table S3 |** The selected parameters for the fitted likelihood distribution functions in the *mid*-*term* future (2040-2069).

| Station | Season | Distribution | Parameters’ Description |
| --- | --- | --- | --- |
| Gamasiab | Spring | 3-Parameter Weibull | Scale: 2304.67675; Shape: 1789.28287; Threshold: -2294.80447 |
|  | Summer | Normal | μ (Location): 24.99844; σ (Scale): 0.88125 |
|  | Autumn | Lognormal | μ (Location): 2.47752; σ (Scale): 0.07707 |
|  | Winter | Normal | μ (Location):-2.55839; σ (Scale): 2.11129 |
| Gharesou | Spring | 3-Parameter Weibull | Scale: 79.58646; Shape: 63.96224; Threshold: -67.63199 |
|  | Summer | Normal | μ (Location): 26.85522; σ (Scale): 0.96691 |
|  | Autumn | Normal | μ (Location): 13.85205; σ (Scale): 0.97223 |
|  | Winter | Normal | μ (Location): 0.03892; σ (Scale): 1.65005 |
| Karkheh-e-Jonobi | Spring | 3-Parameter Weibull | Scale: 10.15078; Shape: 8.37452; Threshold: 13.36723 |
|  | Summer | Normal | μ (Location): 37.64637; σ (Scale): 0.78604 |
|  | Autumn | Lognormal | μ (Location): 3.18962; σ (Scale): 0.04169 |
|  | Winter | 3-Parameter Weibull | Scale: 1.89261; Shape: 1.75725; Threshold: 8.28747 |
| Kashkan | Spring | 3-Parameter Weibull | Scale: 12.44553; Shape: 9.72646; Threshold: 1.97891 |
|  | Summer | Normal | μ (Location): 29.37075; σ (Scale): 0.83547 |
|  | Autumn | Lognormal | μ (Location): 2.79145; σ (Scale): 0.05879 |
|  | Winter | 3-Parameter Weibull | Scale: 2.74164; Shape: 3.28716; Threshold: -0.05814 |
| Seimareh | Spring | 3-Parameter Weibull | Scale: 19.42898; Shape: 15.87098; Threshold: -6.89326 |
|  | Summer | Normal | μ (Location): 27.46528; σ (Scale): 0.90229 |
|  | Autumn | Normal | μ (Location): 14.46045; σ (Scale): 0.97169 |
|  | Winter | Normal | μ (Location): 0.96591; σ (Scale): 1.38328 |

**Table S4 |** The selected parameters for the fitted likelihood distribution functions in the long-term future (2070-2099).

| Station | Season | Distribution | Parameters’ Description |
| --- | --- | --- | --- |
| Gamasiab | Spring | Normal | μ (Location): 11.50229; σ (Scale): 1.19065 |
|  | Summer | Normal | μ (Location): 27.28762; σ (Scale): 0.85589 |
|  | Autumn | Normal | μ (Location): 13.81339; σ (Scale): 0.94935 |
|  | Winter | 3-Parameter Weibull | Scale: 521.37314; Shape: 396.29550; Threshold: -519.91972 |
| Gharesou | Spring | Normal | μ (Location): 13.32649; σ (Scale): 1.23954 |
|  | Summer | Normal | μ (Location): 29.17400; σ (Scale): 0.94623 |
|  | Autumn | Normal | μ (Location): 15.69067; σ (Scale): 0.95571 |
|  | Winter | 3-Parameter Weibull | Scale: 519.05270; Shape: 471.03630; Threshold: -515.93702 |
| Karkheh-e-Jonobi | Spring | Normal | μ (Location): 24.86032; σ (Scale): 1.24046 |
|  | Summer | Normal | μ (Location): 39.80444; σ (Scale): 0.80204 |
|  | Autumn | Normal | μ (Location): 26.26586; σ (Scale): 0.95793 |
|  | Winter | Normal | μ (Location): 11.57549; σ (Scale): 1.30542 |
| Kashkan | Spring | Normal | μ (Location): 15.78962; σ (Scale): 1.22737 |
|  | Summer | Normal | μ (Location): 31.58634; σ (Scale): 0.81318 |
|  | Autumn | Normal | μ (Location): 18.24075; σ (Scale): 0.95445 |
|  | Winter | Normal | μ (Location): 4.80091; σ (Scale): 1.18456 |
| Seimareh | Spring | Normal | μ (Location): 13.95122; σ (Scale): 1.17061 |
|  | Summer | Normal | μ (Location): 29.74422; σ (Scale): 0.86800 |
|  | Autumn | Normal | μ (Location): 16.32850; σ (Scale): 0.94292 |
|  | Winter | Normal | μ (Location): 3.12706; σ (Scale): 1.26068 |

**Table S5 |** The selected parameters for the fitted posterior distribution functions in the short-term future (2010-2039).

| Station | Season | Distribution | Parameters’ Description |
| --- | --- | --- | --- |
| Gamasiab | Spring | Normal | μ (Location): 8.55317; σ (Scale): 0.84243 |
|  | Summer | Logistic | μ (Location): 23.13724; σ (Scale): 0.33492 |
|  | Autumn | Lognormal | μ (Location): 2.36659; σ (Scale): 0.06971 |
|  | Winter | Gamma | Scale: 0.29289; Shape: 6.53607 |
| Gharesou | Spring | 3-Parameter Weibull | Scale: 3.80777; Shape: 4.52359; Threshold: +7.46313 |
|  | Summer | 3-Parameter Weibull | Scale: 2.25401; Shape: 3.16593; Threshold: +23.08802 |
|  | Autumn | 3-Parameter Weibull | Scale: 4.59912; Shape: 6.63782; Threshold: +8.85786 |
|  | Winter | 3-Parameter Weibull | Scale: 8.70271; Shape: 7.21131; Threshold: -6.85797 |
| Karkheh-e-Jonobi | Spring | 3-Parameter Weibull | Scale: 6.38941; Shape: 7.41667; Threshold: 16.07004 |
|  | Summer | 3-Parameter Weibull | Scale: 5.20181; Shape: 10.29238; Threshold: 31.00639 |
|  | Autumn | 3-Parameter Weibull | Scale: 3.63207; Shape: 4.29469; Threshold: 19.60270 |
|  | Winter | Gamma | Scale: 0.10972; Shape: 86.53337 |
| Kashkan | Spring | Normal | μ (Location): 13.02676; σ (Scale): 1.06369 |
|  | Summer | Normal | μ (Location): 27.2442; σ (Scale): 0.47637 |
|  | Autumn | 3-Parameter Weibull | Scale: 3.32479; Shape: 4.20409; Threshold: 12.14219 |
|  | Winter | Normal | μ (Location): 1.89918; σ (Scale): 1.06046 |
| Seimareh | Spring | Loglogistic | μ (Location): 2.43101; σ (Scale): 0.06349 |
|  | Summer | Normal | μ (Location): 25.73505; σ (Scale): 0.63253 |
|  | Autumn | 3-Parameter Weibull | Scale: 2.78162; Shape: 3.60713; Threshold: +10.63898 |
|  | Winter | Weibull | Scale: 1.34225; Shape: 1.97993 |

**Table S6 |** The selected parameters for the fitted posterior distribution functions in the *mid*-*term* future (2040-2069).

| Station | Season | Distribution | Parameters’ Description |
| --- | --- | --- | --- |
| Gamasiab | Spring | 3-Parameter Weibull | Scale: 4.57578; Shape: 4.80158; Threshold: 5.39706 |
|  | Summer | Logistic | μ (Location): 24.59723; σ (Scale): 0.46882 |
|  | Autumn | Gamma | Scale: 0.07407; Shape: 163.95085 |
|  | Winter | 3-Parameter Weibull | Scale: 2.17544; Shape: 2.07818; Threshold: 0.46869 |
| Gharesou | Spring | 3-Parameter Weibull | Scale: 5.27877; Shape: 6.37831; Threshold:7.03382 |
|  | Summer | Lognormal | μ (Location): 3.27002; σ (Scale): 0.03376 |
|  | Autumn | Normal | μ (Location): 14.35010; σ (Scale): 0.77585 |
|  | Winter | 3-Parameter Weibull | Scale: 6.05802; Shape: 5.98843; Threshold: -3.78195 |
| Karkheh-e-Jonobi | Spring | 3-Parameter Weibull | Scale: 14.26127; Shape: 14.42162; Threshold: +9.39712 |
|  | Summer | Logistic | μ (Location): 37.36406; σ (Scale): 0.43496 |
|  | Autumn | Normal | μ (Location): 24.37424; σ (Scale): 1.00472 |
|  | Winter | 3-Parameter Weibull | Scale: 2.65241; Shape: 2.43170; Threshold: 8.42798 |
| Kashkan | Spring | 3-Parameter Weibull | Scale: 4.28520; Shape: 5.08866; Threshold: 10.12064 |
|  | Summer | 3-Parameter Weibull | Scale: 4.42302; Shape: 9.18538; Threshold: 23.55369 |
|  | Autumn | 3-Parameter Weibull | Scale: 3.03830; Shape: 4.10647; Threshold: 13.59799 |
|  | Winter | 3-Parameter Weibull | Scale: 3.54170; Shape: 4.50252; Threshold: -0.45633 |
| Seimareh | Spring | Weibull | Scale: 13.00659; Shape: 12.87748 |
|  | Summer | 3-Parameter Weibull | Scale: 4.02339; Shape: 4.84154; Threshold: 23.53645 |
|  | Autumn | 3-Parameter Weibull | Scale: 5.20316; Shape: 6.29577; Threshold: 9.8226 |
|  | Winter | Weibull | Scale: 2.57460; Shape: 2.36444 |

**Table S7 |** The selected parameters for the fitted posterior distribution functions in the *long*-*term* future (2070-2099).

| Station | Season | Distribution | Parameters’ Description |
| --- | --- | --- | --- |
| Gamasiab | Spring | 3-Parameter Weibull | Scale: 4.20848; Shape: 4.33760; Threshold: 7.12750 |
|  | Summer | Logistic | μ (Location): 26.52899; σ (Scale): 0.46451 |
|  | Autumn | 3-Parameter Weibull | Scale: 3.27147; Shape: 4.11817; Threshold: 10.62382 |
|  | Winter | Weibull | Scale: 2.63728; Shape: 3.97969 |
| Gharesou | Spring | Normal | μ (Location): 12.66739; σ (Scale): 0.71350 |
|  | Summer | Logistic | μ (Location): 28.38242; σ (Scale): 0.56024 |
|  | Autumn | Logistic | μ (Location): 15.37259; σ (Scale): 0.43589 |
|  | Winter | 3-Parameter Weibull | Scale: 10.80044; Shape: 15.65635; Threshold: -7.47992 |
| Karkheh-e-Jonobi | Spring | 3-Parameter Weibull | Scale: 4.50693; Shape: 4.83111; Threshold: 20.34878 |
|  | Summer | Logistic | μ (Location): 39.14603; σ (Scale): 0.44827 |
|  | Autumn | 3-Parameter Weibull | Scale: 4.68337; Shape: 6.14148; Threshold: 21.74225 |
|  | Winter | 3-Parameter Weibull | Scale: 4.07332; Shape: 4.22478; Threshold: 8.19788 |
| Kashkan | Spring | 3-Parameter Weibull | Scale: 4.24993; Shape: 6.03004; Threshold: 10.88135 |
|  | Summer | Normal | μ (Location): 28.54001; σ (Scale): 0.42219 |
|  | Autumn | 3-Parameter Weibull | Scale: 5.01478; Shape: 7.43513; Threshold: 12.88136 |
|  | Winter | 3-Parameter Weibull | Scale: 3.76894; Shape: 4.41179; Threshold: 1.37289 |
| Seimareh | Spring | Gamma | Scale: 0.07724; Shape: 178.95652 |
|  | Summer | Logistic | μ (Location): 29.08498; σ (Scale): 0.48731 |
|  | Autumn | 3-Parameter Weibull | Scale: 3.12935; Shape: 3.72821; Threshold: 13.59703 |
|  | Winter | Weibull | Scale: 3.70962; Shape: 3.22293 |

**Table S8 |** The stochastic behavior of *μ* in the short-term.

| Station | Season | Distribution | Parameters’ Description |
| --- | --- | --- | --- |
| Gamasiab | Spring | Logistic | μ (Location): 8.56216; σ (Scale): 0.04348 |
|  | Summer | Normal | μ (Location): 23.10660; σ (Scale): 0.04225 |
|  | Autumn | Normal | μ (Location): 10.68178; σ (Scale): 0.0562 |
|  | Winter | 3-Parameter Weibull | Scale: 0.19622; Shape: 2.68561; Threshold: +1.81364 |
| Gharesou | Spring | 3-Parameter Weibull | Scale: 0.67385; Shape: 13.42694; Threshold: +10.18875 |
|  | Summer | Lognormal | μ (Location): 3.22224; σ (Scale): 0.00187 |
|  | Autumn | Lognormal | μ (Location): 2.58099; σ (Scale): 0.00458 |
|  | Winter | Weibull | Scale: 1.12445; Shape: 6.96874 |
| Karkheh-e-Jonobi | Spring | Normal | μ (Location): 22.10940; σ (Scale): 0.10092 |
|  | Summer | Normal | μ (Location): 35.93642; σ (Scale): 0.03907 |
|  | Autumn | Normal | μ (Location): 22.91562; σ (Scale): 0.07861 |
|  | Winter | 3-Parameter Weibull | Scale: 0.36576; Shape: 4.94179; Threshold: +9.23559 |
| Kashkan | Spring | 3-Parameter Weibull | Scale: 0.43500; Shape: 4.28015; Threshold: +12.72852 |
|  | Summer | Normal | μ (Location): 27.26958; σ (Scale): 0.02747 |
|  | Autumn | Logistic | μ (Location): 15.16919; σ (Scale): 0.03579 |
|  | Winter | Normal | μ (Location): 1.93381; σ (Scale): 0.11181 |
| Seimareh | Spring | Normal | μ (Location): 11.15112; σ (Scale): 0.13330 |
|  | Summer | Lognormal | μ (Location): 3.24814; σ (Scale): 0.00161 |
|  | Autumn | Lognormal | μ (Location): 2.58275; σ (Scale): 0.00493 |
|  | Winter | Normal | μ (Location): 1.25348; σ (Scale): 0.04458 |

**Table S9 |** The stochastic behavior of *μ* in the *mid*-*term* condition.

| Station | Season | Distribution | Parameters’ Description |
| --- | --- | --- | --- |
| Gamasiab | Spring | Loglogistic | μ (Location): 2.26274; σ (Scale): 0.00524 |
|  | Summer | 3-Parameter Weibull | Scale: 0.27743; Shape: 4.42983; Threshold: 24.36244 |
|  | Autumn | Normal | μ (Location): 12.06899; σ (Scale): 0.08510 |
|  | Winter | 3-Parameter Weibull | Scale: 0.22826; Shape: 2.58504; Threshold: 2.30781 |
| Gharesou | Spring | 3-Parameter Weibull | Scale: 0.30072; Shape: 4.25121; Threshold: 11.72180 |
|  | Summer | Lognormal | μ (Location): 3.27064; σ (Scale): 0.00267 |
|  | Autumn | Lognormal | μ (Location): 2.66412; σ (Scale): 0.00355 |
|  | Winter | Normal | μ (Location): 1.89635; σ (Scale): 0.11118 |
| Karkheh-e-Jonobi | Spring | Normal | μ (Location): 23.21261; σ (Scale): 0.10957 |
|  | Summer | 3-Parameter Weibull | Scale: 0.15446; Shape: 2.93967; Threshold: 37.18396 |
|  | Autumn | 3-Parameter Weibull | Scale: 0.21725; Shape: 2.58818; Threshold: 24.19938 |
|  | Winter | Lognormal | μ (Location): 2.36990; σ (Scale): 0.00876 |
| Kashkan | Spring | 3-Parameter Weibull | Scale: 0.82648; Shape:10.86683; Threshold: 13.21185 |
|  | Summer | 3-Parameter Weibull | Scale: 0.07797; Shape: 2.08833; Threshold: 27.72254 |
|  | Autumn | Lognormal | μ (Location): 2.79320; σ (Scale): 0.00357 |
|  | Winter | Lognormal | μ (Location): 1.01980; σ (Scale): 0.02278 |
| Seimareh | Spring | Lognormal | μ (Location): 2.51427; σ (Scale): 0.00930 |
|  | Summer | Lognormal | μ (Location): 3.30527; σ (Scale): 0.00240 |
|  | Autumn | Lognormal | μ (Location): 2.68899; σ (Scale): 0.00558 |
|  | Winter | Lognormal | μ (Location): 0.76529; σ (Scale): 0.04236 |

**Table S10 |** The stochastic behavior of *μ* in the *long*-*term* condition.

| Station | Season | Distribution | Parameters’ Description |
| --- | --- | --- | --- |
| Gamasiab | Spring | Gamma | Scale: 0.00078; Shape: 14139.15254 |
|  | Summer | 3-Parameter Weibull | Scale: 0.29721; Shape: 5.55366; Threshold: +26.19243 |
|  | Autumn | Normal | μ (Location): 13.63062; σ (Scale): 0.07906 |
|  | Winter | Normal | μ (Location): 2.40516; σ (Scale): 0.05086 |
| Gharesou | Spring | Normal | μ (Location): 12.54233; σ (Scale): 0.06161 |
|  | Summer | Normal | μ (Location): 28.37247; σ (Scale): 0.08390 |
|  | Autumn | 3-Parameter Weibull | Scale: 0.31709; Shape: 5.82222; Threshold: +15.12518 |
|  | Winter | Normal | μ (Location): 3.01822; σ (Scale): 0.05770 |
| Karkheh-e-Jonobi | Spring | Lognormal | μ (Location): 3.19627; σ (Scale): 0.00340 |
|  | Summer | Normal | μ (Location): 39.03591; σ (Scale): 0.05610 |
|  | Autumn | Normal | μ (Location): 26.03975; σ (Scale): 0.07015 |
|  | Winter | Normal | μ (Location): 12.02088; σ (Scale): 0.09937 |
| Kashkan | Spring | Normal | μ (Location): 14.84005; σ (Scale): 0.06474 |
|  | Summer | Normal | μ (Location): 28.52252; σ (Scale): 0.02960 |
|  | Autumn | Normal | μ (Location): 17.57622; σ (Scale): 0.04946 |
|  | Winter | Normal | μ (Location): 4.78858; σ (Scale): 0.08409 |
| Seimareh | Spring | Lognormal | μ (Location): 2.62419; σ (Scale): 0.00693 |
|  | Summer | Lognormal | μ (Location): 3.37023; σ (Scale): 0.00196 |
|  | Autumn | Logistic | μ (Location): 16.36099; σ (Scale): 0.03719 |
|  | Winter | Normal | μ (Location): 3.36215; σ (Scale): 0.10258 |
